# Proton beam range verification by means of ionoacoustic measurements at clinically relevant doses using a correlation-based evaluation

**DOI:** 10.3389/fonc.2022.925542

**Published:** 2022-11-03

**Authors:** Jannis Schauer, Hans-Peter Wieser, Yuanhui Huang, Heinrich Ruser, Julie Lascaud, Matthias Würl, Andriy Chmyrov, Marie Vidal, Joel Herault, Vasilis Ntziachristos, Walter Assmann, Katia Parodi, Günther Dollinger

**Affiliations:** ^1^ Institute for Applied Physics and Metrology, Bundeswehr University Munich, Neubiberg, Germany; ^2^ Faculty of Physics, Chair of Medical and Experimental Physics, Ludwig-Maximilians-University, München, Germany; ^3^ Institute of Biological and Medical Imaging (IBMI), Helmholtz Zentrum München, Neuherberg, Germany; ^4^ Chair of Biological Imaging for Translational Cancer Research (TranslaTUM), School of Medicine, Technical University of Munich, Munich, Germany; ^5^ Centre Antoine Lacassagne (CAL), Department of Radiation Oncology, Nice, France

**Keywords:** ionoacoustics, protoacoustics, proton therapy, *in-vivo* range verification, range verification in proton therapy, cross-correlation, matched filtering, signal-to-noise ratio

## Abstract

**Purpose:**

The Bragg peak located at the end of the ion beam range is one of the main advantages of ion beam therapy compared to X-Ray radiotherapy. However, verifying the exact position of the Bragg peak within the patient online is a major challenge. The goal of this work was to achieve submillimeter proton beam range verification for pulsed proton beams of an energy of up to 220 MeV using ionoacoustics for a clinically relevant dose deposition of typically 2 Gy per fraction by i) using optimal proton beam characteristics for ionoacoustic signal generation and ii) improved signal detection by correlating the signal with simulated filter templates.

**Methods:**

A water tank was irradiated with a preclinical 20 MeV proton beam using different pulse durations ranging from 50 ns up to 1 μs in order to maximise the signal-to-noise ratio (SNR) of ionoacoustic signals. The ionoacoustic signals were measured using a piezo-electric ultrasound transducer in the MHz frequency range. The signals were filtered using a cross correlation-based signal processing algorithm utilizing simulated templates, which enhances the SNR of the recorded signals. The range of the protons is evaluated by extracting the time of flight (ToF) of the ionoacoustic signals and compared to simulations from a Monte Carlo dose engine (FLUKA).

**Results:**

Optimised SNR of 28.0 ± 10.6 is obtained at a beam current of 4.5 μA and a pulse duration of 130 ns at a total peak dose deposition of 0.5 Gy. Evaluated ranges coincide with Monte Carlo simulations better than 0.1 mm at an absolute range of 4.21 mm. Higher beam energies require longer proton pulse durations for optimised signal generation. Using the correlation-based post-processing filter a SNR of 17.8 ± 5.5 is obtained for 220 MeV protons at a total peak dose deposition of 1.3 Gy. For this clinically relevant dose deposition and proton beam energy, submillimeter range verification was achieved at an absolute range of 303 mm in water.

**Conclusion:**

Optimal proton pulse durations ensure an ideal trade-off between maximising the ionoacoustic amplitude and minimising dose deposition. In combination with a correlation-based post-processing evaluation algorithm, a reasonable SNR can be achieved at low dose levels putting clinical applications for online proton or ion beam range verification into reach.

## 1 Introduction and purpose

Compared to X-Ray tumor therapy, ion beam therapy, in particular proton therapy, offers the advantage of a spatially localized dose deposition and therewith reduces the integral dose delivered to the healthy tissue of the patient by a factor 2 to 3 (1, 2) while offering a comparable or even better tumor coverage. This advantage results from the interaction of charged particles with matter. As ions slow down along their track, their stopping power increases until they stop completely and transfer most of their energy at the so-called Bragg peak, behind which a pronounced negative dose gradient occurs. The Bragg peak location can be precisely steered by the initial particle kinetic energy. However, an imprecise knowledge of the traversed integral tissue stopping powers within the patient compromises the accuracy. This imperfect knowledge can be traced back, among others, to anatomical changes introducing discrepancies between the patient anatomy considered for treatment planning and the one actually being irradiated (3) or to the inaccurate calculation of the stopping powers from the CT image used for treatment planning. Range uncertainties become most problematic for ion beams stopping at the distal edge of the tumor with an organ at risk closely behind.

To compensate uncertainties caused by planning and delivery, it is clinical practice to intentionally irradiate a larger volume known as planning target volume (PTV) encompassing the tumor and additional safety margins introduced to ensure with confidence a high and confined dose level within the clinical target volume (CTV). In the advent of high precision radiation therapy not only a safer but also a more aggressive, so-called dose escalation treatment is desirable, but currently hindered by the imprecise knowledge of the exact Bragg peak locations. This is a well-known problem in the medical physics community and studied by various groups (2, 4, 5). A brief excerpt is given next.

Besides robust treatment planning approaches (6), imaging techniques e.g. dual-energy CT (7) or proton radiography/tomography (5, 8) are under investigation to improve pre-treatment proton range predictions. Complementary, an *in-vivo* treatment verification method - ideally in real time for clinically relevant dose levels - would allow precise range adjustments, mitigating problems related to range uncertainties. As all protons stop inside the patient, current *in-vivo* range verification approaches rely on correlating the proton range to secondary emissions generated by the impinging protons.

Positron emission tomography (9) is based on the radioactive decay of radionuclides produced in non-elastic nuclear interactions between the tissue and the incoming protons. The resulting activity pattern deduced from the measurement of coincident 511 keV photons resulting from the annihilation of the positron emitted in the *β* ^+^-decay, offers a typically retrospective evaluation of the stopping location of the protons. This technique depends on the half-life times of the positron emitting radioactive isotopes which lie, depending on the isotope, between milliseconds and minutes. During this time the isotopes can be delocalized due to perfusion and other processes, which results in a so-called washout-effect compromising the interpretation of the signal (9).

A second more direct approach, also relying on nuclear interactions, is given by the detection of MeV prompt photons (4). Here, the excited target nuclei rapidly (< ns) emit characteristic photons which are referred to as prompt gammas. The detection of prompt gammas reveals information about the ion beam range with an accuracy of one millimeter under clinical conditions in a homogeneous phantom (10).

A major challenge for both methods lies in the fact that the signal generation depends on the nuclear reaction threshold and is thus not predominantly generated at the Bragg peak but rather along the total beam path. In addition, the registration of the gammas is performed in the coordinate system of the detector which makes a direct mapping to the patients anatomy error-prone as potential movements of the patient have to be monitored additionally.

A third approach for *in-vivo* range verification is given by ionoacoustics which is based on the emission of an acoustic wave due to local energy deposition (11) caused by a pulsed ion beam. Ionoacoustics offers the possibility of a submillimeter range verification using a time-of-flight (ToF) method (12). The obtained range can be correlated to an ultrasound image of the underlying anatomy as shown in an ex-vivo sample study by Kellnberger et al. (13) or a phantom-based study by Patch et.al (14).

One of the main challenges in the field of ionoacoustics is the poor signal-to-noise ratio (SNR) of the recorded signals considering realistic clinical fractionated dose levels of 1.5 Gy - 3 Gy (15). This problem of low SNR can be tackled in multiple ways: i) improved sensing hardware (e.g. highly sensitive low noise detectors, low-noise amplifiers), ii) usage of optimally suited beam settings including beam current and pulse shape profiles and through iii) signal (post)-processing.

In this study we investigate the dependency of the ionoacoustic signal SNR on the proton pulse duration, the beam current and optimised post-processing filtering. The goal of this study was to achieve precise range verification of the Bragg peak location with a dose deposition of 2 Gy or less. This is realized by optimising the beam characteristics and the post-processing for signals generated by 20 MeV and 220 MeV proton beams outlining an impact for the clinical use case.

## 2 Materials and methods

### 2.1 Experimental setup

For the findings concerning the clinical beam energies, data recorded in 2017 at a clinical synchrocyclotron was re-evaluated. A detailed description of the underlying setup can be found in Lehrack et al. (15).

**Figure 1:**
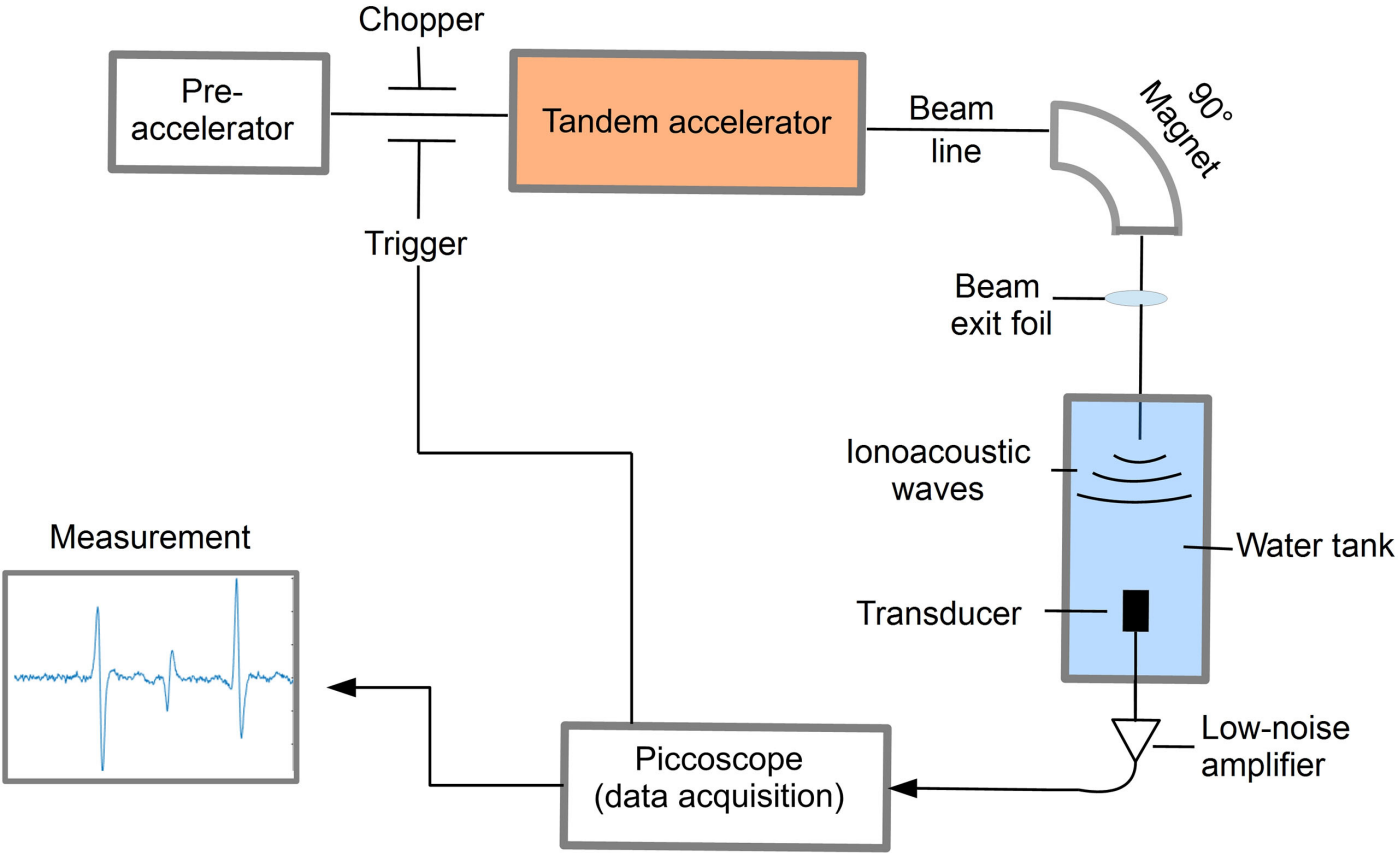
Schematic illustration of the experimental setup to detect ionoacoustic signals at a proton energy of 20 MeV.

For the preclinical experiments, different excitation pulse profiles for ionoacoustic signal generation have been investigated at the 14 MV tandem accelerator of the Maier-Leibnitz-Laboratory of LMU and TUM in Garching near Munich using the setup shown in **Figure 1**.

A beam of 20 MeV protons was used with an instantaneous beam current of up to several μA. The range of these protons in water is 4.21 mm according to Monte Carlo simulations using FLUKA (version 2020.03), modelling the entire beam line and experimental setup (16, 17). Using a slit system the beam size was reduced to a close to rectangular size of 2.5 mm × 3 mm before exiting the vacuum beam line through a 11.4 μm thick titanium foil and entering a water tank through a 50 μm thick kapton foil. The water tank (33 × 18 × 19 cm^3^) was filled with de-ionized water at room temperature. The temperature was recorded alongside every ionoacoustic measurement to ensure a correct calculation of the speed of sound for data evaluation.

To record the ionoacoustic signals an Olympus immersion transducer (Type V382-SU) was mounted on a motorized x-y-z-stage and aligned with the proton beam in axial configuration in preceding measurements. The transducer has a diameter of 12.7 mm and is spherically focused to 25.4 mm, which is ideally its distance to the Bragg peak. The transducer has a central frequency of 3.5 MHz and a fractional bandwidth at -6 dB of 72% (from 2.2 MHz to 4.7 MHz). The transducer signal was first amplified using a 60 dB low noise amplifier (HVA-10M-60-B, FEMTO) before being digitized using a 6404D Picoscope at a sampling rate of 156 MS/s.

**Figure 2:**
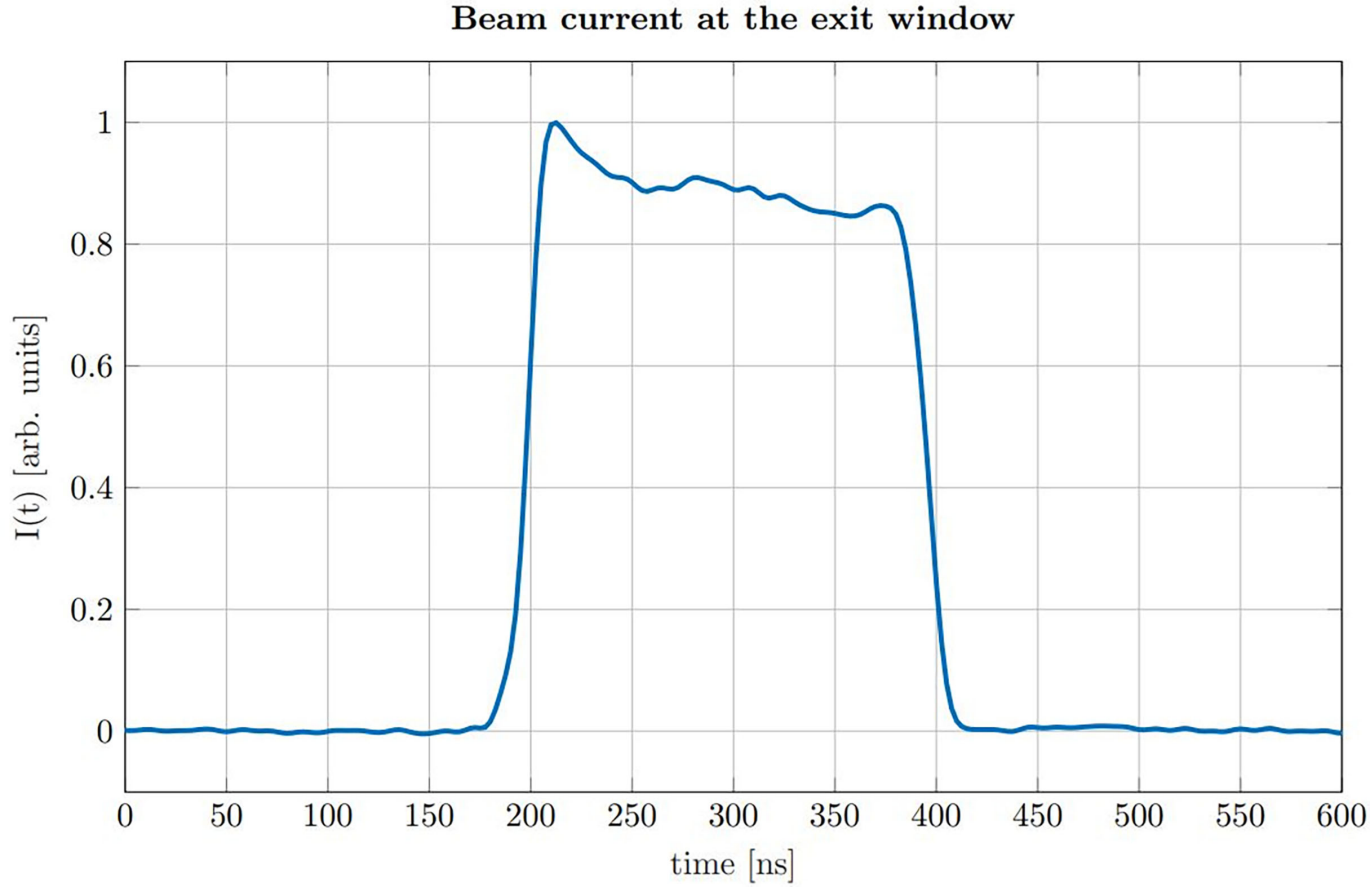
Beam current *I*(*t*) measured at the location of our experimental setup for a nominal pulse duration of 200 ns.

The proton pulses were generated by the low energy chopper system of the tandem accelerator which was driven externally using an Agilent 33220A function generator. The chopper (ideally) delivers rectangular proton pulses, which were directly measured at the beam exit window in a separate experiment beforehand using a fast silicon detector (18, 19). In post-processing the impulse response of the detector was deconvolved from the measured proton time profiles (20). A representative measurement of the time dependent beam current *I*(*t*) for a proton pulse of a nominal duration of 200 ns is shown in **Figure 2**.

The measurement shows that the rise time (from 10% to 90%) is roughly 15 ns, which is short compared to the duration of the pulse (FWHM = 193 ns). The plateau region of the beam current shows minor fluctuations which are small compared to the absolute amplitude and thus validating the approximation of a rectangular pulse shape. The chopper signal was also used as a trigger for data acquisition. The underlying 10 kHz repetition frequency of the chopper allowed to consecutively measure 1000 ionoacoustic signals within 100 ms. Individual measurements were then used for signal averaging during post-processing.

### 2.2 Post-processing

The recorded measurements were post-processed using a cross-correlation filter also known as matched filter. A matched filter maximises the SNR of a measurement for a known signal shape (21). The ideal filter (or template) *T*(*t*) is identical to the signal without noise. The resulting correlation function maximises SNR and peaks at the position of best overlap of the template and the signal. For discretised signals a normalized correlation *CC_M,T_* (*τ*) can be expressed as:


(1) $$CC_{M,T}(\tau )\frac{\Sigma_{t}M(t)\cdot T(t+\tau )}{\sqrt{AC_{M,M}(0)\cdot AC_{T,T}(0)}}$$

Here *M*(*t*) is the measured signal, *T*(*t*) is the template and *τ* is the time shift between the two functions. The normalization in the denominator consists of the auto-correlation functions of the measurement *AC_M,M_* (0) and the template *AC_T,T_* (0) at zero lag and ensures the range of correlation values between -1 and 1.

The templates used in this study were simulated following the theoretical description from Jones et al. (22, 23). The ionoacoustic pressure wave arises from two different components - the temporal heating function *H_t_* (*t*) and the spatial heating function *H_s_* (*r'*, *θ'*, *ϕ'*), which are given by the energy converted per unit time and space, respectively. *H_t_* (*t*) is given by the normalized beam current $H_{t}(t)=\frac{I(t)}{\int I(t)dt}$ in units $\left[\frac{1}{s}\right]$ while $H_{s}$ (*r'*, *θ'*, *ϕ'*) = *ρD*(*r'*, *θ'*, *ϕ'*) is the three dimensional energy density in units $\left[\frac{J}{m^{3}}\right]$ in the coordinate system of the transducer (*r'*, *θ'*, *ϕ'*), which is composed of the dose *D*(*r'*, *θ'*, *ϕ'*) and the mass density *ρ* for the medium in which the dose is deposited. The pressure at the position of the transducer at time *t* is given by the convolution of the temporal heating function $H_{t}$ (*t*) with the core of the spatial heating function $P_{\delta }$ (*t*):


(2) $$p(t)=\frac{\partial }{\partial t}\int_{-\infty }^{\infty }H_{t}(t-\tau )P_{\delta }(\tau )d\tau =\left[\frac{\partial H_{t}(t)}{\partial t}*P_{\delta }(t)\right]$$

*P_δ_* (*t*) is given by (22)


(3) $$P_{\delta }(t)=\frac{v_{s}\beta }{4\pi C_{p}}\frac{1}{R^{\prime }}\int_{0}^{2\pi }\int_{0}^{\pi }d\theta^{\prime }\;d\phi^{\prime }\;R^{\prime 2}\;sin(\theta^{\prime })\rho D(r^{\prime },\theta^{\prime },\phi^{\prime })$$

Equation 3 can be simplified using the dimensionless Grüneisen parameter $\Gamma =\frac{\beta v_{s}^{2}}{C_{p}}$ , where *β* is the thermal coefficient of volumetric expansion and *C_p_* the specific heat capacity at constant pressure. Further, *R'* = *v_s_t* is the distance from the source to the detector which enables a direct transcription from distance to time and vice versa via the acoustic wave velocity *v_s_* within the medium, here water.


(4) $$P_{\delta }(t)=\frac{t}{4\pi }\int_{0}^{2\pi }\int_{0}^{\pi }d\theta^{\prime }\;d\phi^{\prime }\;sin(\theta^{\prime })\Gamma H_{s}(r^{\prime },\theta^{\prime },\phi^{\prime })$$

Where Γ*H_s_* (*r'*, *θ'*, *ϕ'*) = *p*_0_(*r'*, *θ'*, *ϕ'*) is sometimes referred to as the initial pressure (24). Note, that *D*(*r'*, *θ'*, *ϕ'*) is dependent on the position of the transducer and *P_δ_* (*t*) is therefore calculated for one specific position of the transducer. All measurements in this study were conducted using axial positions of the transducer on the distal end of the Bragg peak. Using equation 2 and 3, the templates were simulated following a three-step process.

#### 2.2.1 3D dose distribution

The 3D dose distribution from the proton beams in water was generated using FLUKA (16, 17) with 50 × 10^6^ particles and an isotropic cartesian scoring of 20 μm for the evaluation of the 20 MeV proton experiment. A similar approach was used for the reevaluation of the signals generated by 220 MeV protons using a cartesian scoring of 450 μm and 12 × 10^6^ particles. Assuming no heat defect, the deposited dose was then multiplied by the mass density 998.1 kg m^-3^ and the Grüneisen parameter (0.108 at 20.1°C) to obtain the initial pressure distribution in Pascal.

#### 2.2.2 Pressure propagation

Next, the initial pressure distribution was transferred into k-wave (version 1.3), a MATLAB (25) toolbox used for propagating acoustic waves (26, 27). For the simulation, a sound speed in water of 1482 ms^-1^ and an idealized point sensor was assumed. The distance to the axially positioned detector was 25.4 mm for the 20 MeV proton case and 75 mm for the 220 MeV protons, which mimics the experimental setups. The simulated pressure assuming a delta-shaped excitation was subsequently convolved with the respective temporal heating function, which are rectangular and of variable width for the 20 MeV proton experiment and Gaussian with an FWHM= 3.7 μs for the reevaluation of the experiments conducted with 220 MeV protons at the clinical synchrocyclotron in Nice.

#### 2.2.3 Detector characteristics

In the last step, the simulated pressure trace was convolved with the transfer function of the detector. For the Olympus immersion transducer this transfer function was approximated by a Butterworth band-pass filter of first order adapted to the -6 dB bandwidth (11, 28) (2.2 MHz - 4.7 MHz). The transfer function for the Cetacean Hydrophone which was used to detect the signals generated by the 220 MeV protons was similarly approximated by a Butterworth filter between 10 kHz – 250 kHz, which is the region of flat frequency response according to the Cetacean C305X specs sheet.

For some simulations shown in this manuscript the simulation parameters have been altered to have a more general result which is not dependent on the used detector or the distance from the Bragg peak to the detector. In particular, the transfer function of the detector has been neglected and the distance to the detector was assumed to be large meaning that the spherical integration in equation 3 is flat in the region of the Bragg peak. These alterations are explicitly stated when the corresponding simulations are shown.

### 2.3 SNR assignment

The standard procedure to determine the measurements quality is the SNR. It is defined as the average signal power divided by the average noise power. For discretised signals the SNR is given as:


(5) $$SNR=\frac{P_{signal}}{P_{noise}}=\frac{\frac{1}{N}\sum_{n=1}^{N}A_{signal}^{2}(n)}{\frac{1}{M}\sum_{m=1}^{M}A_{noise}^{2}(m)}$$

Here, *A_signal_* (*n*) and *A_noise_* (*m*) are the amplitude of the signal and the noise respectively, *n* = 1···*N* are all samples considered for the calculation of the signal power and *m* = 1···*M* are all samples considered for the calculation of the noise power. Note that as the sample size *M* increases, the average noise power does not increase but gives a more accurate estimate of its mean value. The noise region was defined before the arrival of the signal to exclude potential acoustic reflections from the noise power calculations.

In the following, SNR_S_ is used when referring to the SNR of the raw ionoacoustic signals and SNR_C_ is used to describe the SNR of the filtered signals after performing the correlation filter.

## 3 Results

This section is structured as follows: First, the template shape is discussed and the templates for the tandem experiments are shown. In the next step, ionoacoustic emissions from a 20 MeV proton beam are investigated for different pulse durations. The experimental SNR_S_ and SNR_C_ are evaluated and compared to simulation results. From these measurements an ideal pulse duration is deduced, which maximises SNR_S_ and SNR_C_ while keeping the deposited dose constant. The dependency of the ionoacoustic signal amplitude on the beam current is discussed. Range verification is performed with a single shot measurement containing a total dose of 0.5 Gy. To prove the feasibility of ionoacoustic range verification in a clinical scenario this section closes with analysing measurements performed with a clinically relevant energy of 220 MeV.

### 3.1 Temporal structure of the templates

For rectangular pulse shapes, which is a good approximation in our 20 MeV proton experiment, $\frac{\partial H_{t}(t)}{\partial t}$ collapses into two delta-like peaks - a positive one when the beam is turned on (compression) and a negative one when the beam is turned off (rarefaction).

**Figure 3:**
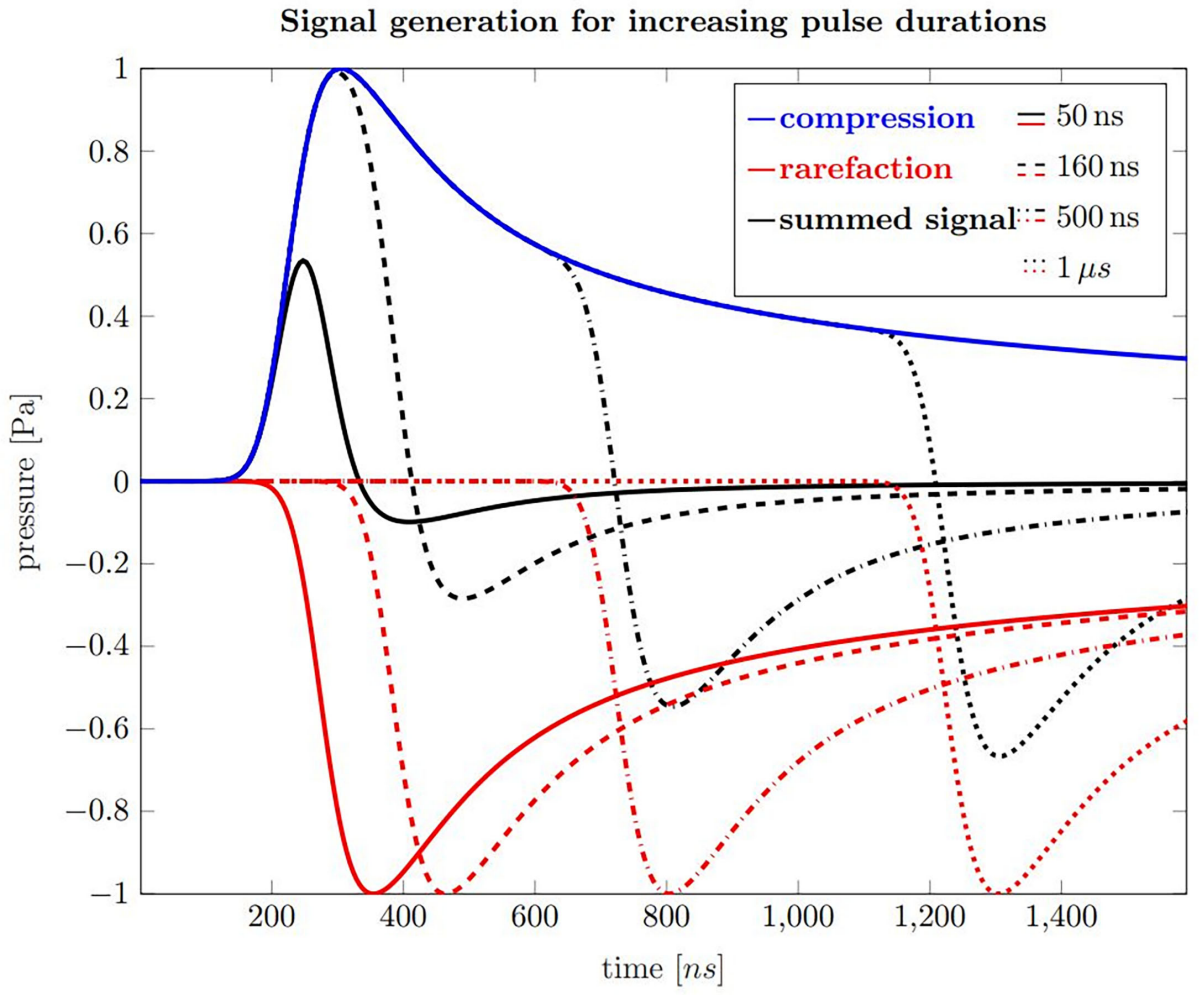
Ionoacoustic signal generation by a rectangular, 20 MeV proton beam as detected by an ideal point sensor. Blue is the compression curve and red are rarefaction curves for pulse durations of 50 ns (solid), 160 ns (dashed), 500 ns (dash-dotted) and 1 µs (dotted). The black curves show the resulting pressure distributions, which arises from the sum of the compression and the corresponding rarefaction curve.

**Figure 3** shows calculations of ionoacoustic signals according to equation 2 generated by varying pulse durations with rectangular shape. The contribution from the temporal heating function can be understood as adding a positive *P_δ_* (*t*) with a shifted negative copy of *P_δ_* (*t*), which are separated by the pulse duration. In order to generate 1 Pa of pressure, the beam current was set to 320 nA. The influence of transducer characteristics on the signal is neglected in these simulations.

The blue curve shown in **Figure 3** is the compression curve that is created when the beam is turned on and the red curves are the rarefaction curves which are generated when the beam is turned off. The delay relative to the compression curve is equal to the pulse durations of 50 ns (solid), 160 ns (dashed), 500 ns (dash-dotted) and 1 μs (dotted), respectively. The sum of the compression and rarefaction curves results in the total temporal pressure distribution at the detector position, shown in black, and represent the ionoacoustic signals to be detected.

Focusing on these signals, the rising slope results from the slope of the spatial heating function when observing the acoustic wave in axial direction distal to the Bragg peak. It is closely related to the slope of the dose profile beyond the Bragg peak. For short pulses up to 160 ns the rarefaction signal destructively interferes with the compression and the overall amplitude is reduced. For pulses longer than that, the maximum amplitude stays unaltered, although the total deposited energy increases. The maximum negative amplitude decreases slightly with increasing pulse duration. The principle form of the acoustic waves does not change when higher proton energies are used but the timescale extends as the Bragg peak becomes wider.

**Figure 4:**
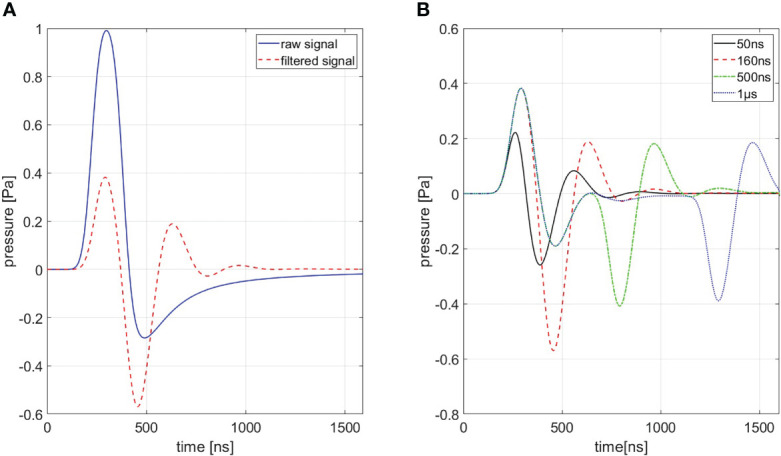
**(A)** Shows a comparison between a signal with (red) and without (blue) transducer characteristics being accounted for a 160 ns rectangular pulse. **(B)** Shows signals of different pulse durations of which transducer characteristics are accounted for. A Butterworth band-pass filter of first order was used as transfer function.

In order to generate a simulation of best conformity, which is needed for the filtering process (cf. *Section 3.2.3*), the transducer characteristics need to be taken into account. These distortions are highlighted in **Figure 4A** for a ionoacoustic signal generated by 20 MeV protons and a rectangular pulse of 160 ns duration. The transducer characteristics influence the amplitude, the frequency content and its phase. **Figure 4B** shows the influence of transducer characteristics for every signal shown in **Figure 3**.

The transducer used to detect the pressure wave distorts the frequency content and the amplitude of the original ionoacoustic signal due to its small bandwidth when compared to the ionoacoustic signal. Frequencies outside the sensitive range of the transducer, in this case especially originating from dose deposition at the Bragg curve entrance, are attenuated.

In addition, the transducers geometrical properties also shape the signal. The sensitive surface area of the transducer can be interpreted as infinitely many point detectors arranged together. A recorded signal can thus be seen as an averaged signal over all point detectors. The measured signal shape and amplitude therefore depend on the position and the angle of the detector relative to the source and the spatial averaging over the surface area.

The effect of spatial averaging can be mitigated with a focused transducer (like in this experiment) which is comprised of a concave sensitive area such that the spherically expanding pressure wave is arriving at the complete detector surface at the same time if the source is at the focal point. Using a focused transducer requires precise positioning of the transducer to keep distortions low. An active change in orientation or a shift of the transducer changes spatial averaging and can consequently have an influence on the recorded signal. All stated effects are modelled by the Butterworth band-pass filter used to approximate the transducer’s transfer function (cf. **Figure 4**). The simulated signals shown in **Figure 4B** have been used as templates for the correlation filtering process (cf. *Section 2.2*).

### 3.2 Signals from single pulses of 20 MeV protons

**Figure 5:**
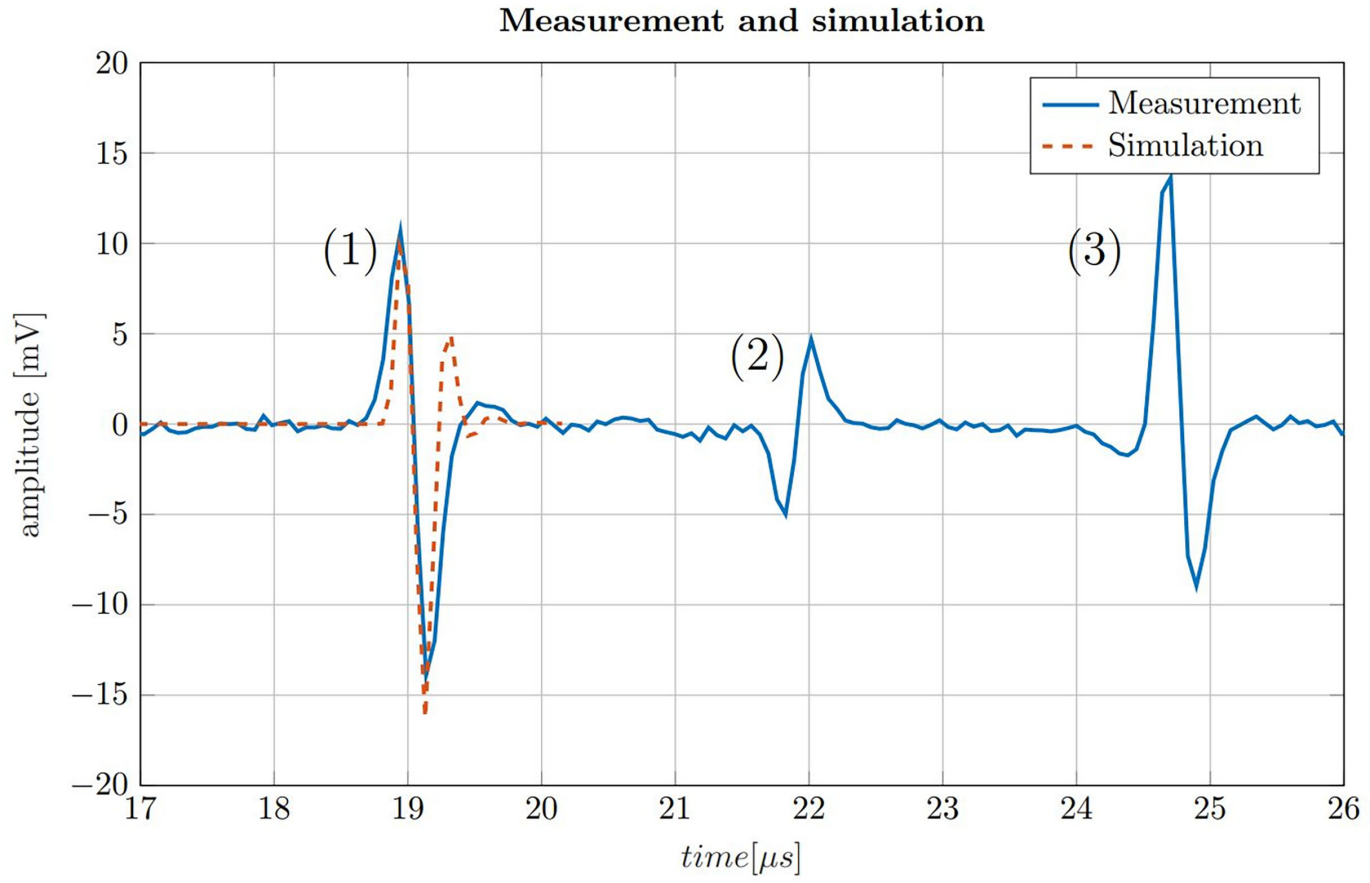
200-fold averaged ionoacoustic signal generated by a 160 *ns* rectangular pulse with a beam current of 4.5 μA and a total integral dose of 128 Gy plotted by the solid blue line. The signal is comprised of (1) the direct signal (2), the window signal and (3) an echo. Additionally a comparison with a simulation is shown for the direct signal by the dashed line, which was scaled to match the amplitude of the signal.

To put the simulations shown in **Figure 4B** in perspective, a ionoacoustic measurement with high integral dose (128 Gy peak dose) is shown in **Figure 5**. The signal is generated by 20 MeV protons and a rectangular heating function of 160 ns duration. The simulation from **Figure 4A** is plotted in addition for a better comparison.

The measurement in **Figure 5** shows three distinct signals. The first signal (1) is called the direct signal. It is caused by the ultrasound waves traveling from their origin at the Bragg peak to the transducer. This is the type of signal whose structure was discussed in **Figure 3**. The direct signal is the signal which is referred to in the following if not stated differently. The second signal (2) is called the window signal which is caused by the beam entering the water tank through the entrance window separating air and water. The third signal (3) is the reflection signal which is a mirrored and phase inverted copy of the direct signal. It is caused by the ultrasound waves traveling backwards from the Bragg peak and being reflected at the entrance window of the water tank.

In addition **Figure 5** shows the similarity between the measurement and the simulation of the direct signal. The simulation has a slightly higher frequency content and overestimates the second maximum of the signal. A possible reason for this dissimilarity are potential misalignments of the transducer from the ideal axial position with zero angular offset. This deviation could be corrected by adjusting the filter boundaries of the Butterworth band-pass filter. However, in realistic applications the signal shape is generally not known in such detail and thus the simulation is therefore not being adjusted according to the measurement. In a clinical scenario, the simulation must already be determined in advance e.g. from a treatment plan, consisting exclusively of information known before the measurement is recorded.

**Figure 6:**
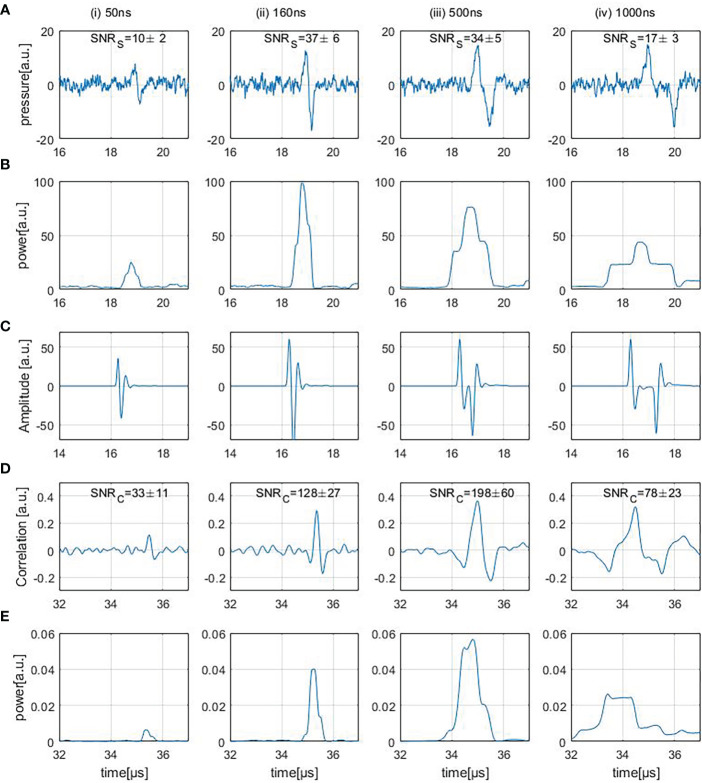
Row **(A)** shows ionoacoustic measurements generated by varying pulse durations. The presented SNR_S_ values are obtained from the moving average power spectra of the raw measurements, shown in row **(B)**. Row **(C)** shows the templates used to filter the measurements [row**(A)**] resulting in the correlation functions [row **(D)**] and row **(E)** shows the moving average power spectra of the correlation functions.

**Figure 6A** shows four different experimentally measured (direct) ionoacoustic signals generated by single rectangular proton pulses of increasing pulse duration and their “moving average power spectra” needed for SNR_S_ calculation in row (B). Row (D) shows the measurement after applying the correlation filter and (E) shows the moving average power spectra of the correlation. The details of each row are discussed in the following.

#### 3.2.1 Signal analysis

Four different ionoacoustic measurements generated by 20 MeV protons and pulse durations of 50 ns, 160 ns, 500 ns and 1 μs at a beam current of 4.5 μA are plotted in **Figure 6A**. Shown are 5 averages each, which corresponds to a peak dose value at the Bragg peak between 1 Gy and 20 Gy linearly depending on the pulse duration according to FLUKA Monte Carlo simulations. The signal amplitude almost doubles between 50 ns and 160 ns while it only increases negligibly for higher pulse durations, which is consistent with the simulations shown in **Figures 3**, **4B**.

#### 3.2.2 SNR_S_ determination

**Figure 6B** shows the corresponding moving average power spectra of the signals of **Figure 6A**. The moving average power spectra are needed for the SNR_S_ assignment of the measurements (cf. Eq. 5) and are calculated as follows.

The duration *d* of the signal was determined using the simulated signals shown in **Figure 4B** avoiding a dependency on noise fluctuations. A threshold of 20% of the maximum amplitude within the rising edge of the signal was determined as the starting point of the signal. To determine the endpoint of the signal, a 20% threshold of the minimum amplitude occurring after the minimum of the signal was used. The expected duration of the signal could then be determined by the difference of these time instants. The average power over that period of time was calculated for every possible location within the measured time frame.


(6) $$P(p)=\frac{1}{d}\sum_{i=p}^{p+d}A_{signal}^{2}(i)$$

*P*(*p*) is the moving average power spectrum and *A_signal_* is the amplitude of the signal. *P*(*p*) peaks when the time interval perfectly overlays with the signal position (cf. **Figure 6B**). From this moving average power spectrum the average signal power can be extracted as the maximum value. The average noise power is determined as the average power in the ionoacoustic signal [row(A)] before the arrival of the signal (at approx. 19 μs).

The interval taken into account for the calculation of the noise power was chosen to end before the arrival of the first signal to exclude possible secondary signals and reflections. Further, the duration of that interval was maximised within the measured time frame ensuring the most accurate estimate of the average noise power. The SNR_S_ depicted in **Figure 6A** increase between the first 2 signals, which is due to the increase in amplitude, and then decreases again. The decrease originates from the fact that the signal generated by a 500 ns pulse is longer but not higher in amplitude than for the 160 ns case and so the average signal power drops.

#### 3.2.3 Correlation filter

A correlation-based evaluation was used to filter and denoise the measurements. The templates used for the filtering process are shown in **Figure 6C**. The resulting filtered signals are shown in row (D). Being a simulation of the expected signal, the template T contains all the known information about the signal shape. Such a filter allows to optimally harvest prior information by means of (in-silico) simulations performed upfront (21).

For every possible time shift *τ* of the template relative to the measurement, the correlation function *CC*(*τ*) is calculated. This correlation function maximises at the location where the signal part of the measurement and the template overlap best, giving rise to the information where the signal can be found within the measurement (**Figure 6D**). Compared to the measurements, the correlation functions show a linearly shifted time axis. The shift (approx. 16 μs) depends on the signal position in the templates [cf. row (C)].

From **Figure 5** it is known that an imprecise knowledge of the signal shape can compromise the accuracy of the template. A way to improve the performance of the correlation further is to use a measured template obtained from a high dose measurement performed beforehand. Such an ideal template would further improve the SNR_C_ of the correlations by ~ 10% compared to the used simulated templates. Since for this procedure the signal shape would need to be known beforehand, this manuscript works with simulated templates only, sacrificing the additional 10% of SNR_C_.

#### 3.2.4 SNR_C_ of correlated signals

The SNR_C_ values of the correlation functions shown in **Figure 6D** were calculated similarly to the SNR_S_ of the measurements as described in *Section 3.2.2.* The moving average power spectra of the correlation functions are needed for the calculation of the SNR_C_ (**Figure 6E**). The duration of the signal was determined by auto-correlating the template and choosing the adjacent zero-crossings next to the main peak as the starting point and end point of the signal. Since **Figure 6E** displays the mean power of the correlation function, its maximum value describes the average power contained in the correlation peak. The average noise power was calculated as the average power contained in the correlation function [row (D)] before the arrival of the correlation peak. To minimize the influence of beam current fluctuations, the displayed SNR_C_ values in **Figure 6D** are mean values which were calculated after repeating the SNR_C_ calculation procedure 100 times and averaging the 100 SNR_C_.

The SNR_C_ increases more than 4-fold from 50 ns to 160 ns which is due to the increase in amplitude within the underlying signals. Between 160 ns and 500 ns there is an additional increase in SNR_C_ even though the unfiltered measurements show a slight decrease in SNR_S_ The reason for the SNR_C_ increase is the fact that the maximum possible SNR_C_ of a correlation function depends on the total energy of the input signal (21). Therefore the SNR_C_ of the correlation functions increases since the signal energy in the 500 ns case increased compared to the 160 ns case. For the correlation function of the 1 μs case the SNR_C_ drops which is mainly due to the fact that the lower frequencies within the underlying measurements cause the correlation peak to broaden and its average power is reduced.

### 3.3 Ideal pulse duration

**Figure 7:**
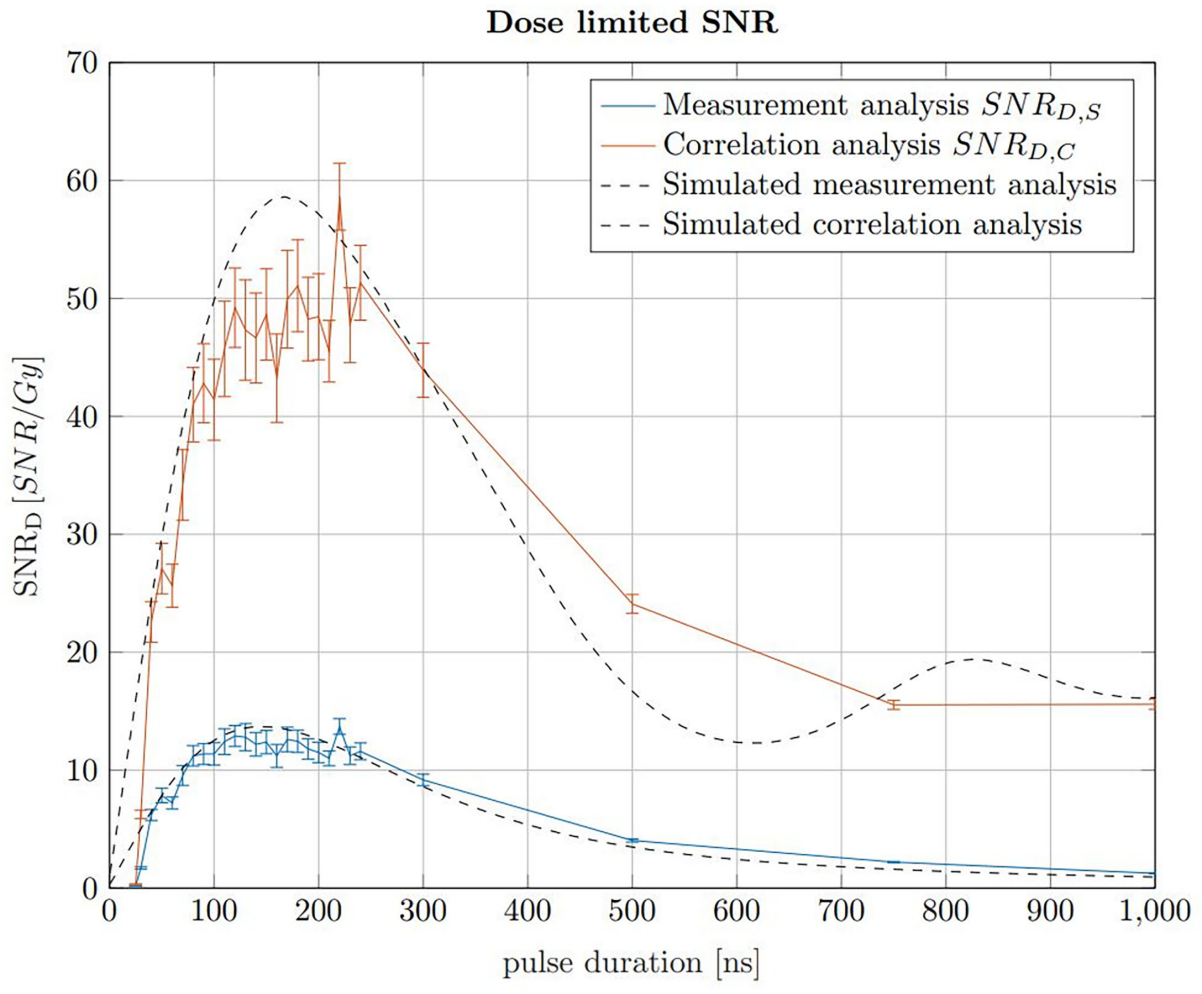
SNR_D,S_ of raw signals in blue and the SNR_D,C_ of the corresponding correlations in red for 20 MeV protons and a constant instantaneous beam current (4.5 μA). The error bars are given as the SEM after 20 independent evaluations. The black curves show simulations of the measured data, which have been scaled to match the measured data.

The pulse duration influences the applied dose, the SNR_S_ and the SNR_C_. The dose increases linearly with the pulse duration assuming a constant instantaneous beam current for all pulse durations. The maximum applicable beam current is assumed to be constant for any pulse duration. In a clinical context, the applicable dose is predefined in the treatment planning process. In contrast to the dose, the SNR increases rapidly with pulse duration for short pulse durations before reaching maximum within a plateau region and declining for longer pulse durations. The ideal pulse duration, which is found for a maximised SNR at a constant dose limit, can be found using a dose limited $SNR_{D}=\frac{SNR}{D}$ . The maximum of this dose limited SNR describes the ideal trade off between applied dose and SNR. In Jones et al. (22), the SNR_D_ was simulated for a given detector position and pulse shape, however here simulations are additionally compared with measurements and the ideal pulse duration is evaluated in the context of the correlation filter. To determine the ideal pulse duration for ionoacoustic signal generation, the SNR_D_ values of signals generated with constant beam current and varying pulse durations recorded in an axial measurement position are shown in **Figure 7**. Here, the beam current was 4.5 μA, however a different beam current would not change the maximum position and therefore the ideal pulse duration but only the SNR_D_.

The SNR_D,S_ (blue) and SNR_D,C_ (red) were calculated by determining the SNR of 200 averages of raw measurements and correlations, respectively, and then dividing by the total dose they contain at the Bragg peak depending on the pulse duration. A similar result is obtained if, with increasing pulse duration, correspondingly fewer measurements or correlations were averaged to meet a dose limit of 1 Gy. For a fair SNR_D_ comparison an experimental noise measurement was used for the noise power calculation. This measurement was performed between two ionoacoustic measurements when the beam was turned off but under otherwise identical conditions and serves as a noise basis for all plotted measurement points. For the red curve (SNR_D,C_) the noise power was calculated after correlating the templates with this experimental noise measurement.

Considering the measured data using 20 MeV protons, **Figure 7** shows that a broad maximum of SNR_D_ is obtained ranging from 100 ns to 230 ns. The error bars are calculated as the standard error of the mean (SEM) and are obtained since each measurement for a given pulse duration was repeated 20 times. The uncertainties are mainly due to beam current fluctuations. However, the simulations in **Figure 7** show that even with idealised conditions a broad spectrum of pulse durations will deliver comparable SNR_D_. There is a certain freedom of choice regarding the pulse duration without diminishing the overall SNR_D_ significantly.

For the simulations in **Figure 7**, ionoacoustic signals including transducer specific properties (templates) were simulated and their average signal power was evaluated. The noise power was assumed to be independent of the pulse duration and therefore constant. Its magnitude was chosen such that the simulation best matches the measured data. For the correlation analysis the simulated signals were auto-correlated and the average signal power of the auto-correlation functions was obtained as signal power. For the noise power calculation the templates were correlated with the experimental noise measurement. This was necessary since the average noise power of the correlation functions is proportional to the contained signal energy of the template.

The improvement between the SNR of the raw measurements and the correlations, $\frac{SNR_{D,C}}{SNR_{D,S}}$ , increases continuously from a factor 3 for short pulse durations (e.g. 50 ns) up to a factor 6 for long pulse durations (e.g. 500 ns). This increase is directly proportional to the contained signal energy of the raw measurements, which continuously increases up to the longest investigated pulse duration of 1 μs. The dependency on the total signal energy also causes the slight shift of the ideal pulse duration of the simulations - from 150 ns when considering only the raw measurements up to 170 ns for the correlations.

### 3.4 Beam current dependency

The preceding section discussed the maximum SNR of ionoacoustic signals and the corresponding pulse duration for a dose limited measurement with fixed instantaneous beam current. However, if the beam current is a free parameter, SNR can be elevated by increasing the beam current (29). The total dose deposition can be kept constant by reducing the number of measurements taken into account for averaging.

**Figure 8:**
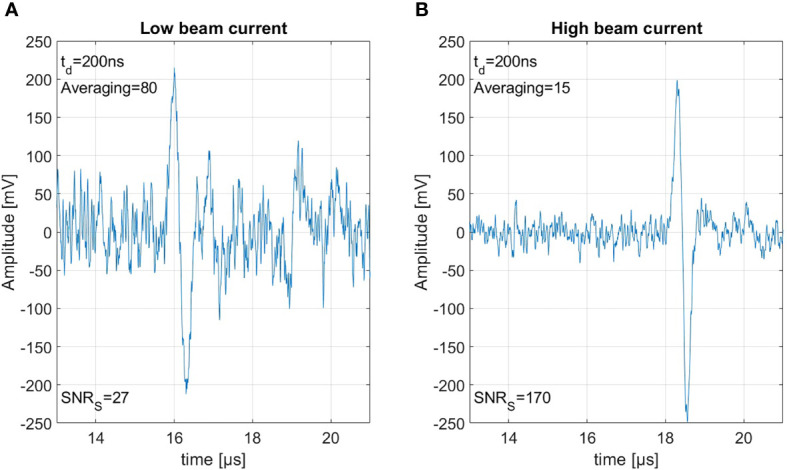
Both measurements were generated using 20 MeV protons, stopping in water. Panel **(A)** shows 80 averaged measurement with 0.15 Gy per shot and panel **(B)** shows 15 averages with 0.8 Gy each, thus based on the same total dose of 12 Gy.

**Figure 8A** shows 80 summed measurements with a beam current of 0.65 μA resulting in a dose of 0.15 Gy per shot, and therefore an accumulated peak dose of 12 Gy. **Figure 8B** shows 15 summed measurements with a beam current of 4.5 μA resulting in 0.8 Gy per shot accumulating the same integral peak dose of 12 Gy. Between the recording of the two measurements, a realignment process was necessary, which includes the repositioning of the detector and a new alignment of the beam. This is on the one hand the reason that the signals arrive slightly delayed due to slightly different detector positions, and on the other hand that due to minimal changes in the beam spot size the ratio of current and dose is not perfectly identical. The measurements here have been summed rather than averaged to show the similar signal amplitude but the much larger noise power from 80 signals compared to the 15 signals. Due to the higher beam current the SNR_S_ could be improved from 27 to an SNR_S_ of 170. This SNR_S_ increase through increasing the beam current directly translates to the corresponding SNR_C_. For both of the measurements shown in **Figure 8** the correlation would again improve the SNR_S_ by roughly a factor of 3.5. This clearly shows that a high beam current is beneficial for ionoacoustic signal generation under the constraint of a given dose limit.

The reason for this increase in SNR is the following: the amplitude of the ionoacoustic signal is directly proportional to the number of particles per shot and thus directly proportional to the beam current. The noise however is not affected by the beam current. Doubling the number of particles in a measurement by increasing the beam current will thus lead to an increased amplitude by a factor of 2 and therefore an increased SNR_S_ by a factor 4 since the SNR_S_ is calculated from the moving average power spectra which includes squaring. Summing two measurements on the other hand will also increase the included number of particles and the ionoacoustic amplitude by a factor of 2. Due to the statistic nature of noise however, the noise floor will increase by the square root of the number of measurements averaged (22). Doubling the number of particles by summing two measurements which only differ in noise will thus increase the relative amplitudes of the signal and the noise by a factor of $\sqrt{2}$ and therefore only increase the SNR_S_ by a factor 2.

Comparing two sets of measurements which are recorded with different beam currents but averaged according to an upper dose limit (cf. **Figure 8**), it is expected that the increase in SNR_S_ can be directly calculated from the ratio of the beam currents (or doses for equal beam spot size). For our case $\frac{SNR_{S}(4.5)}{SNR_{S}(0.65)}=\frac{170}{27}\approx 6.3\approx \frac{4.5}{0.65}$ . Deviations from this equation can originate from beam current instabilities. This beam current dependency which favors high beam currents can be scaled up to the limiting case where the entire dose is delivered in a single shot.

**Figure 9:**
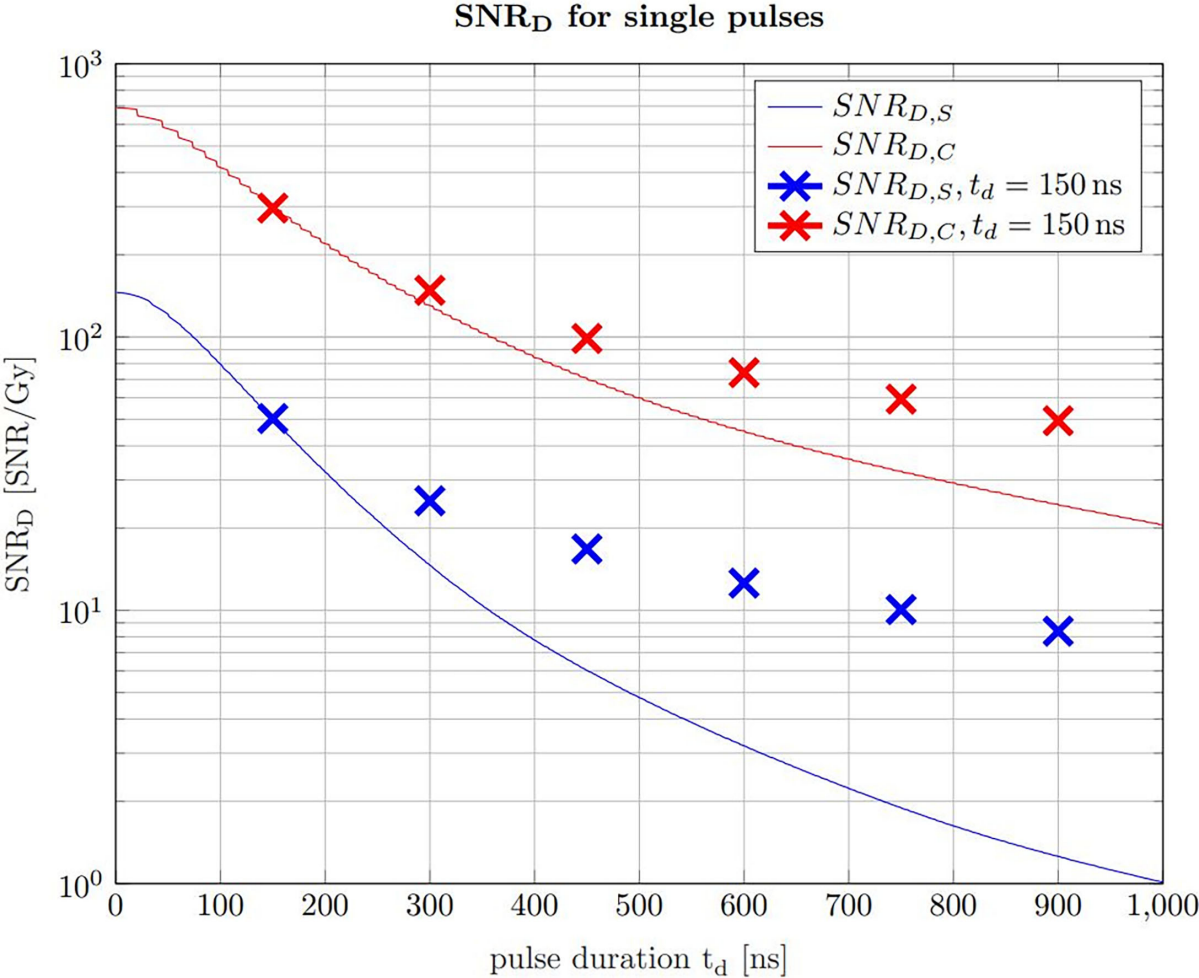
The SNR_D,S_ (solid blue line) and the SNR_D,C_ (solid red line) for non averaged signals at a given dose limit of 1 Gy. With increasing pulse duration, the beam current decreases correspondingly to ensure the constant dose limit. The crosses indicate the SNR_D,S/C_ that is expected using a pulse duration of 150 ns at the same beam current as the single pulses and use averaging to ensure the dose limit.

Starting from a single shot of ideal pulse duration (cf. *Section 3.3*) the question arises if a further reduction in pulse duration is useful in order to enable an even higher beam current at a constant dose. **Figure 9** shows a comparison of the SNR_D,S_ and SNR_D,C_ of non averaged signals generated by increasing pulse durations and variable beam current to match the given dose limit (solid lines). For these simulations transducer characteristics have been neglected and a dose limit of 1 Gy was assumed. Further, Gaussian pulse shapes were assumed since for very short pulse durations the approximation of infinitely short rise times within a rectangular pulse fails and would distort the results.

The solid lines show the SNR_D,S_ and the SNR_D,C_ for a variable beam current, which means that for progressively shorter pulse durations the beam current is increased to match the given dose limit of 1 Gy. The crosses indicate the expected SNR_D,S_ and SNR_D,C_ at a pulse duration of 150 ns and an averaging number which matches the given dose limit. The beam current along a vertical line is thus always constant and the single pulse indicated by the solid line is chopped in 150 ns pulses and averaged for the SNR_D_ values indicated by the crosses. **Figure 9** shows that for pulses exceeding the ideal pulse duration found in **Figure 7**, it is more efficient to chop the pulses at 150 ns and average the resulting multiple signals. It also shows that the higher the beam current is, the higher is the expected SNR_D,S_ and SNR_D,C_ Starting from a single pulse of 150 ns duration, **Figure 9** shows that a further reduction in pulse duration and a complementary increase of the beam current leads to even higher SNR_D_ values until the limiting case where the whole dose is applied in the shortest time possible.

### 3.5 Dose and range estimate

**Figure 10:**
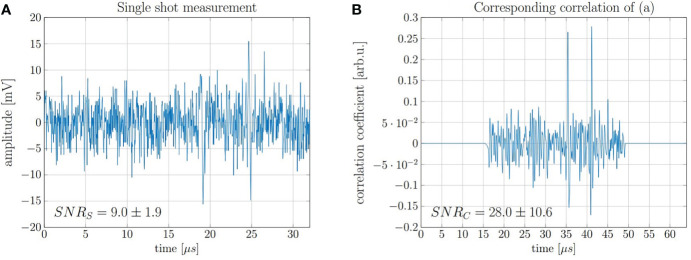
**(A)** Measurement of a single 130 ns pulse with high beam current and a dose deposition corresponding to 0.5 Gy and **(B)** the corresponding correlation function.

In order to demonstrate accurate range determination for the 20 MeV protons at low doses, the final range evaluation was performed with idealized conditions with regard to beam current and pulse duration. It was sufficient to limit the evaluation on a single shot measurement of a 130 ns proton pulse at a beam current of 4.5 μA which corresponds to ~ 3.7 × 10^6^ protons per shot and thus a total peak dose of 0.5 Gy. A representative single shot measurement and the corresponding correlation function obtained after filtering with the simulated template are shown in **Figure 10**. The displayed SNR_S_ and SNR_C_ are average values after evaluating every of the 200 independently recorded measurements.

The range of the protons was evaluated using the direct signal and its reflection (cf. **Figure 5**), which are the two signals visible in **Figure 10B**. Since the reflected signal is travelling backwards from the Bragg peak and is being reflected at the entrance window, its delay compared to the direct signal (ToF) approximates the time needed for the ultrasound waves to travel twice the range of the proton beam. This ToF was determined by calculating the temporal offset between the correlation peak of the direct signals and the reflection signal. The speed of sound in water at the measurement temperature (22.4°*C*) is *v_s_* = 1482 m/s.


(7) $$R=\frac{v_{s}\times ToF}{2}$$

The range *R* was calculated for 200 independent measurements. The mean value of the measured ranges is 4.25 mm which is 40 μm or ≈1*%* off when compared with FLUKA Monte Carlo simulations (4.21 mm). From the 200 individually evaluated measurements a standard deviation of the individual measurements of 0.01 mm was found which corresponds to a relative standard deviation of 0.25% of the full range. This corresponds to a standard error of the mean (SEM) of 700 nm and a relative SEM of 0.02%. However, the FLUKA simulated range shows a systematic deviation > 3*σ*. The reason for the systematic offset could be in the accuracy of the simulation or in a systematic error within the evaluation process. One possible systematic error could be that the phase difference between the direct and the reflected signal is not exactly 180° as assumed in the evaluation. However, this effect is not further evaluated. Nonetheless, the measured ranges are in good agreement with the simulation considering the fact that the absolute deviation was below 70 μm for all evaluated measurements.

### 3.6 Transfer to clinically relevant energies

#### 3.6.1 Measurements with 220 MeV

**Figure 11:**
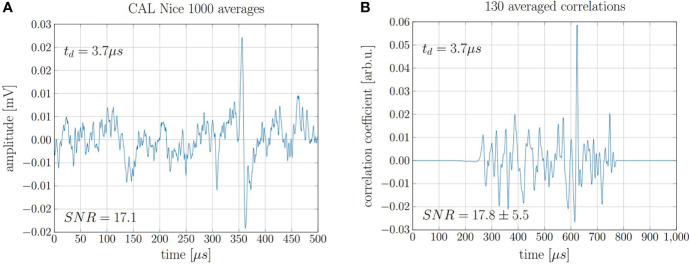
**(A)** 1000 averaged signals depositing 0.01 Gy maximum dose each and **(B)** 130 averaged correlations of individual signals.

The findings for the 20 MeV proton beam can be extrapolated to higher, clinically relevant beam energies. This is proven by the repeated evaluation of measurements recorded in 2017 at the clinical synchrocyclotron of the Centre Antoine Lacassagne (CAL) in Nice, France (15). The exact beam characteristics and the experimental setup can be found in Lehrack et. al (15). The acoustic signal of 220 MeV protons was measured for a pulse with a nearly Gaussian shape of 3.7 μs FWHM. The underlying high-frequency microbunch structure of the beam which is intrinsic to any high frequency accelerator has no significant influence on the signal shape due to the low pass filtering characteristics of the ionoacoustic signals due to the underlying broad dose distribution of the Bragg peak in clinical applications. In this study, where no correlation analysis was applied, 1000 consecutive measurements had to be averaged in order to obtain a submillimeter range verification using a ToF method accumulating a total deposited dose at the Bragg peak of roughly 10 Gy (15). After repeating the 1000-fold measurement five times, a jitter of the maximum position corresponding to a range uncertainty of *σ* = 0.40 mm was found. Applying the cross correlation filter to this data, a similar SNR_C_ could be achieved using only 13% of the measurements and thus reducing the applied dose to a clinically relevant value of 1.3 Gy. A comparison of 1000 averaged raw signals and 130 averaged correlation functions is shown in **Figure 11**.

The evaluation of seven independent measurement sets of 130 single acquisitions each allowed for the evaluation of the fluctuations of the maximum position of the correlation functions. The evaluation of the true range would need a full decomposition including transducer function, temporal heating function etc. However the statistical range uncertainty can be directly assessed from the jitter of the correlation peaks. The jitter was calculated as the standard deviation from the mean peak position and is Δ*t* = 380 ns which corresponds to a range precision of Δ*R* = ± 0.57 mm or ± 0.19% when compared to the range of the protons of 303 mm.

#### 3.6.2 Optimal pulse shape and durations for ionoacoustics in proton therapy

**Figure 12:**
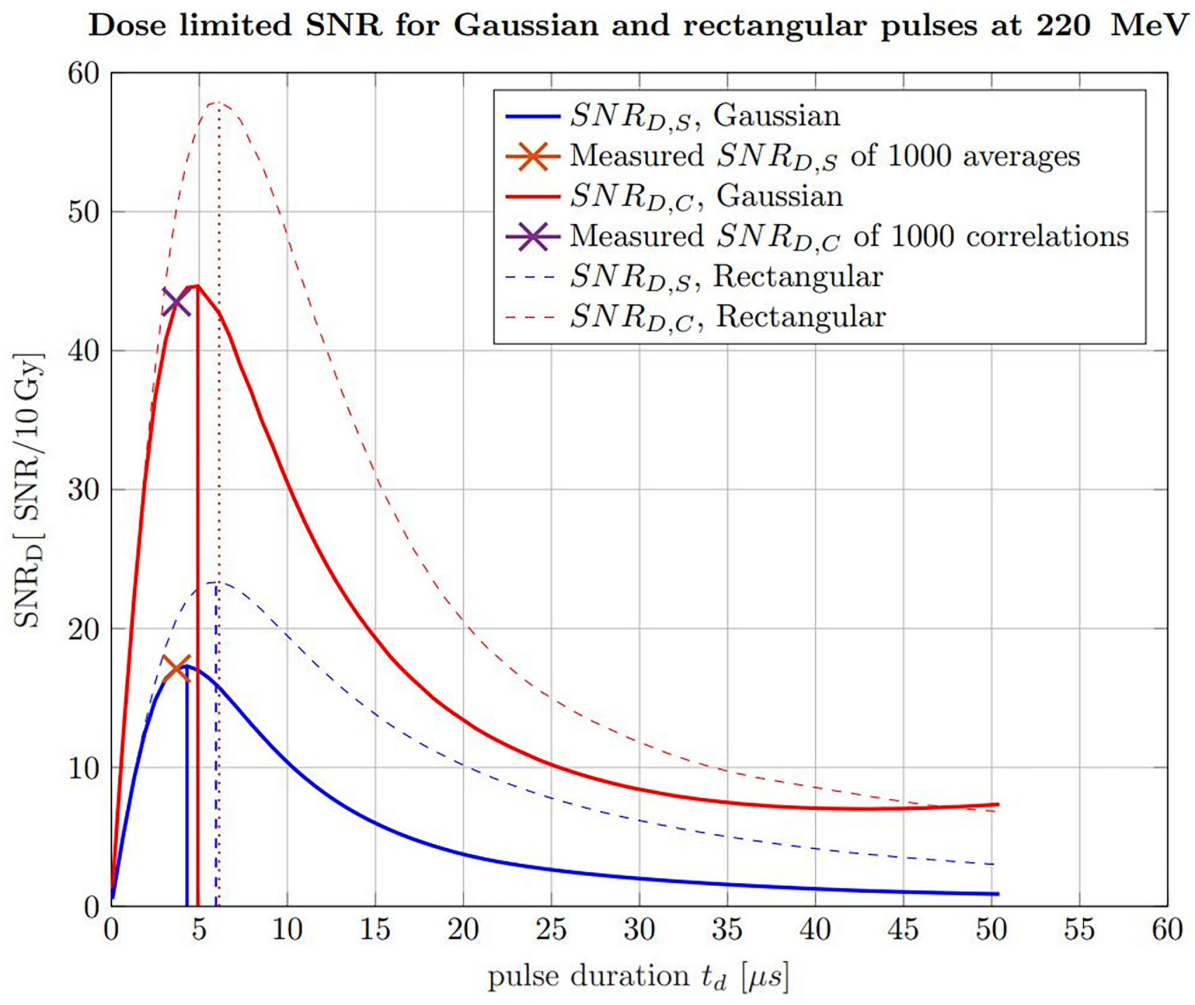
Simulated SNR_D_ of raw ionoacoustic signals for Gaussian (solid) and rectangular (dashed) in blue and their corresponding correlations in red. The measurement analysis done by Lehrack et al. (15) is shown by the orange cross and the corresponding correlation evaluation by the purple cross. Additionally, the maximum position, which indicate the ideal pulse durations are marked by the vertical lines.

Apart from applying the correlation filter, the findings about the ideal pulse duration can also be extrapolated to any proton beam energy being relevant for tumor therapy. The ideal pulse duration can be determined with simulations of ionoacoustic signals generated by increasing pulse durations. This is shown in **Figure 12** for 220 MeV protons including rectangular (dashed) and Gaussian (solid) pulse shapes, for which the pulse duration was chosen to be the FWHM. Similar to **Figure 7**, this has been done considering raw measurements and the corresponding correlation functions in water with a fixed beam current. The calculated signal power is normalized by the integral dose of the given pulse duration to ensure a fair comparison (SNR_D_).

For the raw measurements, the noise power was assumed to be independent from the pulse durations and thus constant. To generate an accurate estimate of the noise power for the correlation functions, the simulated signals for increasing pulse durations have been correlated with experimentally measured noise, following the procedure shown in **Figure 7**. This noise measurement was performed under the same clinical conditions as the ionoacoustic measurement to ensure a realistic and equal noise bases for the simulations. In addition, the curves were scaled, so that the measured SNR_S_ for 1000 averaged signals and SNR_C_ of 1000 averaged correlations could be marked within the simulations. This also defines the total deposited dose and the beam current to be equal with the measurement conditions, which is 10 Gy and 0.43 *μA* instantaneous beam current respectively.

From the straight blue line in **Figure 12** it can be extracted that the FWHM of the pulse, which maximises the SNR_D,S_ at a given dose (here 10 Gy), is 4.3 μs, considering only raw measurements and Gaussian pulse shapes. For a correlation-based evaluation this ideal FWHM increases up to 4.9 µs as indicated by the solid red straight lines.

For the measurements indicated by crosses in **Figure 12**, the used pulse duration of 3.7 µs is shorter than the ideal pulse duration of 4.3 µs for pure signal analysis or 4.9 µs for a correlation analysis. However, because of the flat top of the curve this deviation only accounts for an SNR_D,S_ reduction of 1.2% and a SNR_D,C_ reduction of 2.0% of the maximum amplitude.

Additionally, **Figure 12** shows a comparison between Gaussian pulse shapes (solid lines) and rectangular pulse shapes (dashed lines). It can be seen that the maximum possible SNR_D_ at a given dose is higher if rectangular pulse shapes are used. The reason for this lies in the steep gradients of the temporal heating function which contribute to a more efficient signal generation. Secondly, the ideal pulse durations in the case of rectangular pulse shapes are higher, namely 6.0 µs for raw measurements analysis and 6.1 µs for a correlation-based evaluation.

**Figure 13:**
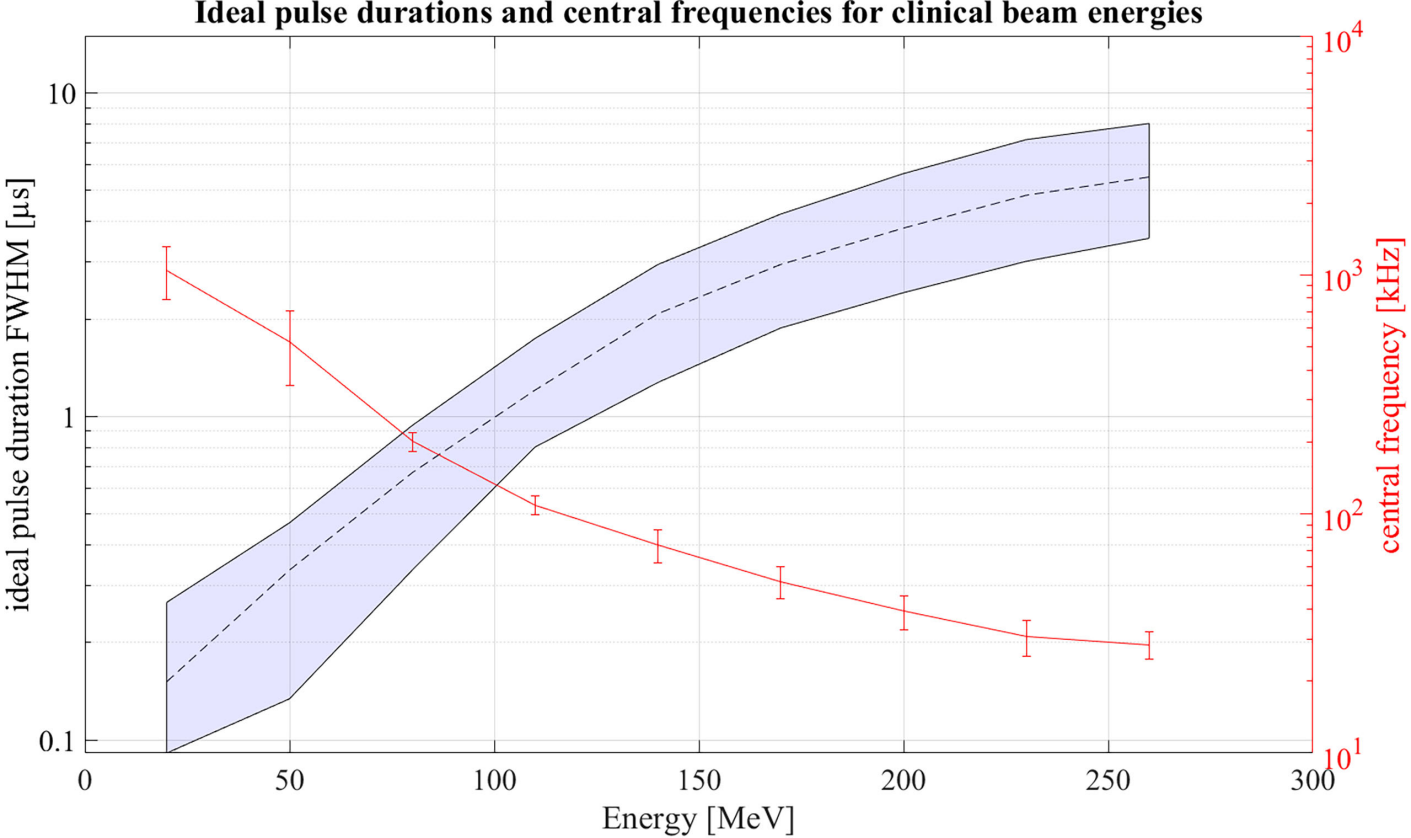
Ideal pulse durations for clinically relevant energies assuming Gaussian pulse shapes. Within the shaded area 90% of the maximum possible SNR_D,C_ can be reached. On the right y-axis the central frequencies of the signals generated with ideal pulse duration is shown.

Extending the simulations further, **Figure 13** shows ideal pulse durations considering Gaussian pulse shapes and a correlation-based evaluation for all proton energies between 20 MeV and 260 MeV. For the simulations a Monte Carlo modelled, mono-energetic proton beam and a broadband point transducer in axial position was assumed. Additionally a large distance from the Bragg peak to the transducer was assumed. Analogue to **Figure 12** measured noise was used in order to generate an accurate noise estimate. The shaded area shows all possible pulse durations for a given energy with which at least 90% of the maximum possible SNR_D,C_ can be reached. Additionally, **Figure 13** shows the expected central frequencies for the signals generated with these ideal pulse durations in red on the right y-axis. The 90% threshold within the ideal pulse duration causes a variance in the possible central frequencies which is depicted by the error bars. Because of the increasing range straggling the Bragg curve flattens and widens for increasing beam energies. This causes the increase of the optimal pulse duration for increasing energies and also shifts the frequency content of the ionoacoustic signal to lower frequencies. Note that the frequency spectrum of a single ionoacoustic signal is broadband, so that also frequency components outside the error bars play an important role for ideal detection. The results shown in **Figure 13** can still vary depending on the used transducer, transducer position and beam parameters.

## 4 Discussion

In this work, ideal beam conditions for maximizing the SNR_S_ of ionoacoustic signals have been presented and evaluated experimentally using a 20 MeV proton research beam. Ideal conditions include rectangular shapes of proton pulses with ideal duration and a maximal beam current. A significant reduction in SNR was found at a constant dose and beam current if a long pulse is applied compared to the application of several pulses of ideal duration.

A correlation-based data analysis method was presented using simulated templates. While in previous work an intensive investigation of ionoacoustic signals in frequency domain was carried out (30), the correlation-based approach showed a simpler, more robust approach with smaller errors in determining the Bragg peak position when comparing for the same dose deposition. It was shown that using this filter, the SNR_S_ could be drastically improved to achieve a statistical range uncertainty of 40 μm (1%) range accuracy for a total dose deposition of 0.5 Gy, which is only a quarter of a typical tumor treatment fraction.

In this preclinical study, ionoacoustic signals were measured with a sensor submerged in water and reflections were used to determine the range of the protons. Considering a more realistic setup with clinical conditions a different approach will be pursued. The measurement of ionoacoustic signals outside a water tank requires the acoustic coupling of the sensor to an irradiated phantom or a patient. Due to the low acoustic impedance of air, a coupling medium such as ultrasonic gel will be introduced between the surface of the phantom and the sensor. The method of range verification also needs to be changed since reflected signals are not assumed to be present with sufficient signal quality. Instead a time of flight method will be used, utilizing e.g. a prompt-*γ*-trigger (15), a transmission detector in front of the patient or an electronic signal from the beam preparation to indicate the starting point and the arrival of the ionoacoustic signal as a stopping point.

The resulting time of flight obtained after evaluating the time interval from trigger to the signal arrival will provide information about the distance from the Bragg peak to the transducer, which is not to be mistaken for the proton range. This information is valuable when it is combined with an imaging device (e.g. ultrasound) positioned at (or right next to) the transducer. For an integration into a treatment planning system, it is necessary to couple both devices (ionoacoustic sensor and ultrasound head) acoustically to the patient’s body. Ionoacoustic signals, ideally recorded in axial position distal to the Bragg peak, can be correlated in real time with already created templates and the extracted Bragg peak position can be marked in the ultrasound image. This would reveal the relative position of the Bragg peak to the tumor or surrounding organs visible in the ultrasound image. It allows an online verification of the Bragg peak position and a possible correction of the treatment plan for measured discrepancies between planned and measured Bragg peak positions. For future clinical applications this is a desirable feature. The proof of concept has been done by Kellnberger et al. (13) and Patch et al. (14) incorporating the Bragg peak location within an ultrasound image using the same device as transmitter and receiver. However, for clinical energies the low-frequency ionoacoustic signal and the high-frequency ultrasound image will likely need to be recorded with different devices, making a spatial co-registration between the ultrasound device and the ionoacoustic detector unavoidable.

Another challenge which will be encountered when pursuing the correlation-based evaluation process are heterogeneities within the patient resulting in multiple signals and reflections. For homogeneous media the simulation of the signals and thus the creation of the templates is well-developed and accurate, making the correlation-based approach straightforward to implement. For heterogeneous materials these simulations are sensitive to the initial conditions e.g. knowledge on the exact location and material properties of the heterogeneities. A possible way to overcome this challenge is to implement self adapting and self optimizing templates based on an initial guess obtained by a simulation. It is also conceivable to implement the information obtained by an ultrasound image directly into the modelling of the template. Another realistic approach looking into the clinical application is to establish a database of signals, to serve as a template for the denoising procedure. A recent study established a dictionary of simulated signals obtained for different detector positions which were then used for a denoising procedure (31). However, without dose limitations, accurate templates could be generated experimentally including information on the proton pulse shape and characteristic transducer responses. These experimentally measured templates could be recorded in homogeneous media or in heterogeneous phantoms consisting of a similar arrangements of materials as in the patient. They could then be correlated with measurements obtained from actual clinical treatment irradiation.

An important component to achieve accurate online range verification is to further improve the SNR of ionoacoustic measurements, e.g. by the increase of the beam current. Irradiation with increased beam currents is already being investigated in the context of FLASH irradiations where side effects are expected to be reduced compared to conventional irradiations at the same dose to the target (32). High beam currents for FLASH irradiation or hypofractionated treatments would therefore also improve online range verification by ionoacoustics.

## 5 Conclusion

This paper explored SNR maximization in ionoacoustic measurements considering beam specific properties and post-processing methods. Optimized beam settings in terms of pulse duration and beam current were theoretically deduced and experimentally verified. In addition, a new correlation-based evaluation was presented making it possible to measure ionoacoustic signals at clinically relevant dose levels, achieving a range verification accuracy of 40 μm for 20 MeV protons when compared to FLUKA Monte Carlo simulations which corresponds to 1% of the total range. For 220 MeV protons a statistical jitter of ± 0.6 mm for was found at a dose of 1.3 Gy, which is below 0.2% of the full range.

The achievement of measuring signals with radiation doses of less than 2 Gy not only with low energy protons but also at clinically relevant energies is making ionoacoustics a promising approach for range verification in ion beam therapy. Additional options to increase the SNR further like an increased beam current and the option to implement a database of prerecorded measurements with high integral dose, which are used as templates for the filtering process, raises legitimate hope to establish ionoacoustics as a standard, non-invasive online range verification method in the future.

## Data availability statement

The raw data supporting the conclusions of this article will be made available by the authors, without undue reservation.

## Author contributions

JS main author, data evaluation and experimentator. H-PW equal contribution as main author. YH help during experiments. HR colleague helping with data evaluation. JL help during experiments especially data acquisition. MW responsible for pulse shape measurement at the tandem accelerator. AC supervisor of YH, present in meetings. MV medical physicist at CAL managing the beam during acquisition of the signals using 220 MeV protons. JH medical physicist at CAL managing the beam during acquisition of the signals using 220 MeV protons. VN leader of institute of YH. WA researcher at LMU, helped with experience in ionoacoustic measurements. KP leader of institute of H-PW. GD leader of institute of JS, major contribution during experiments and writing. All authors contributed to the article and approved the submitted version.

## Funding

The German Research Foundation (DFG, project NR: 403225886) was responsible for funding this project. Other financial support was provided by the EU project Radiate, the Centre for Advanced Laser Applications (CALA) and the Munich-Centre for Advanced Photonics (MAP).

## Acknowledgments

The authors acknowledge the help of the operators of Maier-Leibniz-Laboratory Munich as well the help from the medical physicists at CAL. Special thanks also to Katrin Schnürle for establishing an accurate beam model for the synchrocycloton at CAL. The authors further acknowledge the financial support by DFG (Project NR: 403225886), the EU project Radiate, the Center for Advanced Laser Applications (CALA) and the Munich-Centre for Advanced Photonics (MAP).

## Conflict of interest

The authors declare that the research was conducted in the absence of any commercial or financial relationships that could be construed as a potential conflict of interest.

## Publisher’s note

All claims expressed in this article are solely those of the authors and do not necessarily represent those of their affiliated organizations, or those of the publisher, the editors and the reviewers. Any product that may be evaluated in this article, or claim that may be made by its manufacturer, is not guaranteed or endorsed by the publisher.
